# Troubleshooting ProSeal LMA

**Published:** 2009-08

**Authors:** Bimla Sharma, Jayashree Sood, Chand Sahai, V P Kumra

**Affiliations:** 1,3 Senior Consultant, Department of Anaesthesiology, Pain and Perioperative Medicine, Sir Ganga Ram Hospital, Old Rajinder Nagar, New Delhi-110 060; 2 Senior Consultant, Chairperson, Department of Anaesthesiology, Pain and Perioperative Medicine, Sir Ganga Ram Hospital, Old Rajinder Nagar, New Delhi-110 060; 4 Emeritus Consultant, Chairperson, Department of Anaesthesiology, Pain and Perioperative Medicine, Sir Ganga Ram Hospital, Old Rajinder Nagar, New Delhi-110 060

**Keywords:** Airway management, Equipment, ProSeal laryngeal mask airway, Classic laryngeal mask airway, Troubleshooting

## Abstract

**Summary:**

Supraglottic devices have changed the face of the airway management. These devices have contributed in a big way in airway management especially, in the difficult airway scenario significantly decreasing the pharyngolaryngeal morbidity. There is a plethora of these devices, which has been well matched by their wider acceptance in clinical practice. ProSeal laryngeal mask airway (PLMA) is one such frequently used device employed for spontaneous as well as controlled ventilation. However, the use of PLMA at times maybe associated with certain problems. Some of the problems related with its use are unique while others are akin to the classic laryngeal mask airway (cLMA). However, expertise is needed for its safe and judicious use, correct placement, recognition and management of its various malpositions and complications. The present article describes the tests employed for proper confirmation of placement to assess the ventilatory and the drain tube functions of the mask, diagnosis of various malpositions and the management of these aspects. All these areas have been highlighted under the heading of troubleshooting PLMA. Many problems can be solved by proper patient and procedure selection, maintaining adequate depth of anaesthesia, diagnosis and management of malpositions. Proper fixation of the device and monitoring cuff pressure intraoperatively may bring down the incidence of airway morbidity.

## Introduction

The ProSeal laryngealmask airway (PLMA) is the most complex and advanced version among all the laryngeal mask airways (LMAs).^1^,^2^ Some of the problems with its use are unique, such as oesophageal aspiration of air, gastric distension and airway obstruction which can occur even when the PLMA is correctly placed with a proper insertion technique^3^‐^5^. The other problems encountered are akin to the classic laryngeal mask airway (cLMA), with varying degrees of frequency and intensity. As a routine after insertion and inflation of the PLMA cuff to 60 cm H_2_O, the correct placement of the device is confirmed by several observations and certain specific tests designated to assess PLMA positioning and evaluate the ventilatory and drain tube functions of the mask. These diagnostic tests are simple and quick to perform and the first five of the following are more popular.

1. Visual assessment of depth of insertion
2. Unobstructed inspiratory and expiratory flow
3. Suprasternal notch tap test
4. Gel displacement test
5. Passage of gastric tube/ polyvinyl chloride (PVC) catheter through drain tube
6. Soap bubble test
7. Thread test
8. Self-inflating bulb technique
9. Trachlight™ test
10. Maximum minute ventilation (MMV) test

After confirming correct positioning, the PLMA is properly secured to avoid dislodgement as its cuff is bulkier than that of the cLMA.

### 1. Visual assessment of depth of insertion

Assess for adequate depth of insertion by examining the relation of the integral bite block to the incisors. Ideally the bite block lies between the teeth but protrudes in case the PLMA is inadequately inserted. Stix and O'Connor in a study of 274 adults, found that when the ProSeal LMA was correctly positioned, the midway point of the bite block was proximal to the incisors in 78% of women and 92% of men. A PLMA with its bite block lying entirely outside the mouth is almost unquestionably malpositioned^6^

### 2. Unobstructed inspiratory and expiratory flow

This is assessed by manually ventilating the patient, observing chest movements, capnography, expired tidal volume (V_T_) of > 8m1/kg, and evaluating the compliance by feel of the bag. The reported incidence of airway obstruction with PLMA has been found to vary from 2-10%.^7^,^8^. Increased resistance is suspected with partial obstruction resulting from infolding of the PLMA cuff or downfolding of epiglottis.^3^ The PLMA, with its large drain tube and cuff, may produce respiratory obstruction by displacing the cricoid cartilage anteriorly thereby exerting direct pressure on the arytenoid bodies and muscular processes.^9^

### 3. Suprasternal notch tap test or Brimacombe bounce

The suprasternal notch tap test or the “Brimacombe bounce” confirms the location of the PLMA tip in the oesophagus behind the cricoid cartilage. The test was first described by O'Connor etal in 2002.^10^ It involves tapping the suprasternal notch or cricoid cartilage, and observing simultaneous movement of a column of lubricant, or a soap bubble membrane at the proximal end of the drain tube Both the structures lie in close proximity to the hypopharynx, where the correctly placed distal cuff sits. The drain tube must be patent for the test to be positive. The test works by cuff compression causing drain tube compression within the drain tube, which in turn moves the lubricantor soap bubble. O'Connor etal^10^ reported a low false-negative rate for the suprasternal notch tap test in 50 adults, but false positives and negatives can occur. False positives can occur if the last 1-2 cm of the drain tube is folded over but some of the drain tube is still patent within the distal cuff.^11^ False negatives can occur if the oesophagus is open, since this can weaken the pressure wave.

### 4. Gel Displacement Test

**Fig 1:**
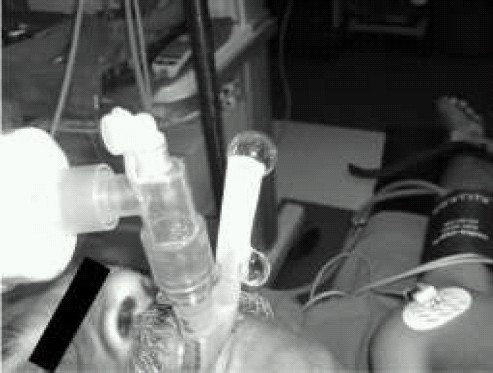
Gel displacement test leaking drain tube

Water-soluble gel (0.5-1 ml) is placed at the proximal end of the drain tube so that it forms a column of about 2-3 cm. Minimal movement or gentle up and down movements indicates a normal position. However, gel ejection with gentle positive pressure ventilation (PPV), indicates a leak from the drain tube, signifying improper seal of device with the hypopharynx (Fig 1). Thus, when positive, the test indicates airway leak through the drain tube.^1^,^2^

### 5. Passage of gastric tube/ PVC catheter through drain tube to verify the patency of drain tube

The posterior folding of the mask tip is ruled out by the successful passage of a gastric tube or a PVC catheter through the drain tube.^1^,^2^,^12^

### 6. Soap Bubble Test

In this test, soap bubble solution is placed over the tip of the drain tube and following observations may be made. When the tip of the PLMA is in the laryn-gopharynx, soap bubble solution column bubbles or the soap membrane bursts during positive pressure ventilation. When the PLMA tip enters the glottis, the tracheobronchial tree communicates directly to the drain tube. The drain tube transmits the airway pressures unless it is obstructed. The PLMA insertion into the glott is is diagnosed by watching either the formation of a spontaneous bubble which is blown away from drain tube port or the soap membrane oscillations seen with cardiac rhythm of the patient.^13^,^14^

### 7. Thread test

A gauze thread or small piece of cotton held over the proximal end of a leaking drain tube can also be used to detect air leak from the drain tube.^13^

### 8. Self-inflating bulb technique

This technique has been used for verification of proper placement of the oesophageal tracheal combitube®.^15^ A self-inflating bag is attached to the drain tube, the bulb injects easily and then remains collapsed with normal positioning of the PLMA. However, during glottic insertion, the self-inflating bulb injects easily and then re-inflates.^16^

### 9. Trachlight™

The Trachlight™ helps in quickly distinguishing glottic from oesophageal location of the tip of the PLMA mask. Trachlight™ (Laerdal Medical, Wappingers Falls, NY, USA) after removing its stylet is passed through the drain tube just as for blind endotracheal intubation.^16^ This is a simple and reliable means of detecting a PLMA tip fold over.^17^ A dull glow in the anterior neck with passage of the Trachlight™ wand beyond the drain tube tip indicates correct alignment of the PLMA with the upper esophageal sphincter.

### 10. Maximum Minute Volume Ventilation (MMV)

The MMV test consists of manually hyperventilating an anaesthetized and paralyzed patient with a PLMA for 15 seconds and extrapolating the total exhaled volume to one minute which can be graded as follows.

**Table:** 

Basal value	5–7 L/min
Critical value	6-12 L/min, threshold for removal of
PLMA	
Mean value	26-29 L/min

The test is easy to perform and can be completed with equipment that is readily accessible to almost every anaesthesiologist.

Anaesthesiologists should be alerted to the potential for significant airway obstruction in any patient with a MMV less than 12 L/min. It is advisable to remove the PLMA and use an alternative device before the initiation of surgery.^9^ In this scenario, one should not have a false sense of security due to the normal oxygen saturation as the latter does not guarantee the satisfactory elimination of CO_2_.^18^ However, the decision to remove the PLMA should be based depending on the patient's physical status, nature, site and duration of surgery.

#### Trouble Shooting

Problems related to the PLMA might occur during: i) insertion of the device ii)maintenance/ emergence phases of anaesthesia iii) recovery phase; in the post anaesthesia care unit or in the ward. Most of the problems are detected in the perioperative period but some airway morbidity and nerve injuries might continue even after the patient has been discharged from the hospital. Various tools required for the purpose of trouble shooting are the PLMA itself with its cuff, drain tube and bite block, pressure gauge to monitor the oropharyngeal seal pressure, cuff pressure monitor, cotton, gauze thread, water soluble gel, and soap solution. Availability of respiratory module and fiberoptic scope can be very helpful in diagnosis and management of various malpositions. Common problems associated with PLMA use are:

#### I. Functional failure

This may result from several factors. The etiology could vary from failure to negotiate the cuff through the oral cavity, various malpositions to mechanical and dynamic causes contributing to airway obstruction inspite of a correctly placed device.^19^

#### A. Device Insertion failure

The first-time and overall insertion failure rate is 14% and 1% respectively.^19^ This phase may be associated with problems such as difficulty in insertion due to the following reasons:

- Disproportionate oral aperture
  Small oral aperture, inability to open mouth fully such as TM joint ankylosis, inappropriate size of the mask and mask not properly deflated before attempting insertion
- Small oral cavity, small pharynx
- Resistance encountered at posteriorpharyngeal wall during insertion
- Short neck
- Light plane of anaesthesia such as coughing, gagging, retching, stridor, hiccup, or biting of device.

## Diagnosis

Inability to negotiate the mask through oral aperture

### Corrective measures 1,2,12,19‐21

- Proper selection of mask
- Lateral approach where the cuff enters the oropharynx from the side of the hard palate.
- Opening the patient's mouth with a laryngoscope followed by insertion of the device
- Gum elastic bougie /fiberoptic insertion, PVC / gastric tube as stents to stiffen the drain tube^12^
- Deepening level of anaesthesia
- Jaw thrust

### B. Gastric tube insertion failure and gastric insufflation

The failure rate for gastric tube insertion is 4%. The most common causes of failure of gastric tube placement are:^19^

- Inadequate lubrication
- Selection of improper size
- Posterior folding of the mask
- Cooled gastric tube^22^

The failure rate for prevention of gastric insufflation during PPV is 0.1% which is similar to the incidence seen with the tracheal tube.^19^

### C. Dislodgement with loss of airway during maintenance phase

The PLMA gets dislodged resulting in loss of airway during the maintenance phase due to light plane of anaesthesia, improper fixation and changes in position e.g. extreme head down position during gynaecologic surgery and laparoscopic procedures. This can be avoided by proper fixation of the device. In the event of intraoperative displacement of the device, a gastric tube left in situ may be very helpful in reinsertion of the device by simply railroading the drain tube over the gastric tube.^23^

### D. Malposition

One of the many advantages of PLMA over other LMA family members is that its malposition can be diagnosed and managed.^20^,^21^ Slight malrotation is more common with the PLMA as compared to the cLMA probably because of residual rotation in the sagittal plane or distortion of glottic geometry.^24^ Several malpositions have been described and the reported incidence is 5-15% at the first attempt.^19^‐^21^ The instruction manual describes three malpositions;^1^,^2^ (i) insufficient insertion depth, (ii) PLMA insertion into the glottis, (iii) PLMA tip folded backwards behind the bowl against the posterior pharyngeal wall.

Presently six malpositions (with approximate incidence) have been described.^25^‐^27^

1. Distal cuffin laryngopharynx (7%)^2^
2. Distal cuff in glottic inlet (3-6%)^2^,^16^
3. Distal cuff folded over (3.4%)^27^
4. Severe epiglottic downfolding(<0.5%)^7^
5. Supraglottic and glottic compression (0.4%)^5^
6. Infolding of cuff (0.6%)^9^

### 1. Distal cuff in laryngopharynx

When the PLMA is not inserted to the desired depth, the distal cuff sits in the laryngopharaynx resulting in protrusion of the bite block.^2^,^6^,^19^,^21^

## Diagnosis

- Bite block protruding
- Soap bubble test positive

## Corrective measure

Further pushing in of the PLMA without colliding with the glottic inlet.^19^,^21^

### 2. Distal cuff in glottic inlet/PLMA insertion into the glottis

When the PLMA takes an anterior path during insertion, the distal cuff collides with the glottic inlet and either remains there or falls back in the laryngopharynx. PLMA entry into the glottis is not uncommon during insertion attempts because of the bulky and flexible mask tip. When the PLMA enters the glottis then the drain tube acts as an extension of the tracheobronchial tree, airway pressures are therefore transmitted through the drain tube and notthe airway tube.^2^,^16^

## Diagnosis

- Thread test
- Gel displacement
- Soap bubble test
- Self-inflating bulb technique
- Trachlight™

### Corrective measures

Correction usually requires reinsertion using a lateral approach, of the gum elastic bougie (GEB) technique. In majority of cases, the reinsertion of the mask is to a noticeably increased depth of insertion. Location of the PLMA tip in the oesophagus behind the cricoid cartilage can be confirmed using the “suprasternalnotch tap test.”^10^ To distinguish between inadequate depth of insertion and glottic impaction, the PLMA can be pushed further inwards: the former will usually be corrected while the latter made worse, with increased airway obstruction or airway protective reflex activation.^19^ O'Connor and Stix have suggested that these malpositions can be distinguished using the soap bubble test.^13^,^14^

### 3. Distal cuff folded over

**Fig 2:**
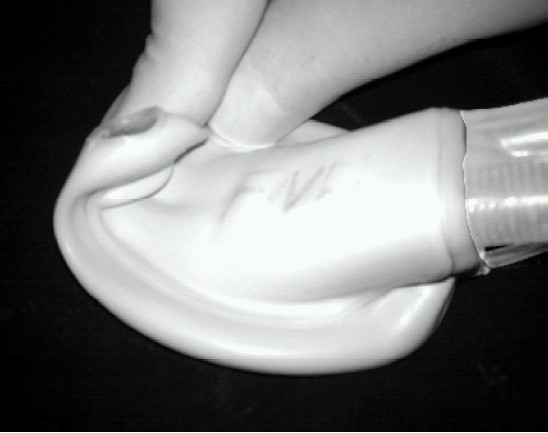
Posterior folding of mask

The advancing distal cuff of the PLMA gets folded (Fig 2) when it impacts against the posterior oropharyngeal wall thereby obliterating the lumen of the drain tube.^19^,^20^ Thus the distal cuff folds up beneath the advancing cuff until the unfolded proximal cuff is redirected inferiorly into the laryngopharynx by the build up of the folded cuff in the oropharynx. The folded distal cuff cannot easily unfold as it gets wedged into the laryngopharynx. Folding over has also been reported with the cLMA,^28^ but is probably more common with the PLMA due to its soft backplate.^26^ This malposition may occur with both finger / introducer insertion and be associated with a better seal and higher mucosal pressures than the correctly placed PLMA.

Brimacom be et al, in a study of 95 patients with the fold over malpositions, found that in 92% resistance was encountered at the back of the mouth, in 83% the bite block protruded from the mouth, and in 98% ventilation was unaffected and the seal was normal.^27^ The main danger of unrecognised fold over phenomenon is that it predisposes the patient to gastric insufflation, regurgitation and pulmonary aspiration as ventilation is unhindered due to easily achieved high airway pressures.^19^ The patency of the drain tube must be assessed in all patients with the PLMA to exclude this malposition. In situations where passage of a gastric tube is not required, the patency can be tested by non-invasively passing the gastric tube or a PVC suction catheter only till the end of the drain tube.

## Diagnosis

- Resistance encountered at the back of the mouth
- Bite block lying outside
- Inability to pass a gastric tube/PVC catheter through the drain tube
- Unaffected ventilation and seal pressure

### Corrective measures^19^‐^21^

- Reinsertion using a lateral approach
- Reinsertion with the drain tube stiffened using a stylet
- Guided insertion with a gum elastic bougie (GEB)
- Digital correction by sweeping a finger behind the cuff

Of these, digital correction appears to be the least effective. Folding over cannot occur with the GEB insertion and gastric tube guided techniques.

### 4. Severe epiglottic downfolding

**Fig 3:**
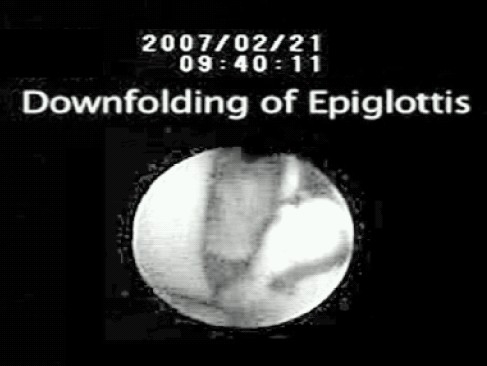
Severe epiglottic downfolding

A well known cause of mechanical airway obstruction is severe epiglottic downfolding which occurs when the epiglottis is dragged inferiorly by the cuff and completely covers the laryngeal inlet (Fig 3). It is diagnosed when the anterior surface of the epiglottis is visible from the airway tube on fiberscope examination.^7^ Although a degree of downfolding of epiglottis has been reported in 17% of cases,^29^ critical airway obstruction seldom occurs from a downfolded epiglottis due to the design feature as the drain tube always suspends the epiglottis off the floor of the bowl. However, with cuff infolding (the two outside cuffs meet in the midline and the epiglottis cannot enter the bowl), a downfolded epiglottis becomes a risk factor for airway obstruction because it is now forced directly on the arytenoids.^9^ It may occur as a result of pre-insertion inflation of cuff, compression of pharynx and enlarged or floppy epiglottis.

## Diagnosis

- High airway pressures
- Airway obstruction
- MMV test
- Fiberoptic examination

### Corrective measures 19‐21

- Reinsertion with the head/neck in a more extreme sniffing position
- Jaw thrust
- Laryngoscope guided placement of PLMA

### 5. Supraglottic and Glottis Compression

Glottic compression occurs when the glottic inlet is mechanically compressed by the distal cuff reducing the tension of the vocal cords.^5^ It is more likely to occur with a small pharynx, over inflated cuff and when the distal cuff is pressed into the hypopharynx with extra force.^19^ Compression of supraglottic and glottic structures may occasionally contribute to significant upper airway obstruction with a correctly placed tip of the cuff lying behind the cricoid cartilage.^9^

## Diagnosis

- High airway pressures
- Airway obstruction
- MMV test
- Fiberoptic examination

### Corrective measures 5,20,21

Reinsertion does not usually solve the problem.

- Air should be withdrawn from the cuff
- Anteroposterior diameter of the pharynx increased by adopting the sniffing position
- Applying jaw thrust

### 6. Cuff infolding

**Fig 4:**
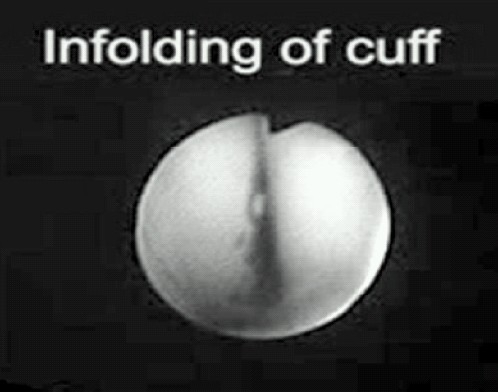
Cuff infolding

Cuffinfolding refers to inward rotation of the large cuffs in front of the bowl so that they contact each other in the midline and obstruct gas flow (Fig 4). It is relatively uncommon and Stix reported 2 cases of cuff in-folding out of 317 cases.^9^,^20^ It is clinically indistinguishable from severe downfolding of epiglottis and both conditions may coexist at times. There is increased risk of cuff infolding with PLMA due to its deeper bowl and a more compliant cuff than that of the cLMA.^2^,^3^

## Diagnosis

- High airway pressures
- Airway obstruction
- MMV test
- Fiberoptic examination

### Corrective measures^20^,^21^

- Sniffing position which increases the anteroposterior diameter of the pharynx
- Applying jaw thrust
- Consider insertion of one size smaller LMA-ProSeal™
- Ensure correct cuff inflation pressures
- Air withdrawal from the cuff may be helpful

Table 1 shows Troubleshooting to various problems, causes, the required confirmatory test and their solutions.

**Table 1:** Troubleshooting ProSeal Laryngeal Mask Airway

Problem	Cause	Confirmatory tests, if any	Solution
(1) • Difficultyin negotiating the cuff at the oral aperture	Disproportionate oral aperture –Small oral aperture –Inappropriate size of the mask –Inability to open mouth fully	• Visual inspection	Insert correct size PLMA Deflate cuff prior to insertion Attempt lateral approach/opening mouth with laryngoscope/ PVC / gastric tube stent for drain tube Laryngoscope used bougie guided insertion
(2) • Insufficient depth of insertion	Disproportionate oral aperture Short neck Light plane of anaesthesia Malposition –PLMA tip in laryngopharynx^1^ – Insertion into glottis 2	Visual inspection Gel displacement Thread test Suprasternal notch test Soap membrane test	Take proper size PLMA Deepen anaesthesia Further pushing in of PLMA will usually correct malposition 1* Removal and reinsertion 2*
(3) • Migration/rotation/ bite block protruding	Overinflation of cuff Herniation of cuff Accidental displacement Posterior folding of mask^3^	Visual inspection - bite block lying outside Inability to pass a gastric tube/PVC catheter through the drain tube Unaffected ventilation and seal pressure	Monitor cuff pressure Pre-insertion cuff integrity checks Proper fixation Lateral approach^3^* Bougie guided insertion 3* Fiberoptic guided, PVC / gastric tube stent for drain tube 3* Remove and reinsert or digitally sweep behind the tip 3*
(4) • Difficultyin passing a gastric tube	Inadequate lubrication/cooled gastric tube Selection of improper size gastric tube Malposition 1‐3 Gross overinflation of cuff	Tactile resistance to insertion Good oropharyngeal seal^3^ Check cuff pressure	Adequate lubrication /warming of tube Proper size selection of gastric tube Correction ofmalposition 1*–3* Monitor cuff pressure
(5) Audible air leak Poor ventilation	Small size of mask Herniation of cuff Inadequate anaesthesia Poor fixation Open upper oesophageal sphincter Malposition^1^	Confirmcuff integrity prior to use; deflate entirely prior to autoclaving Gel displacement test Soap bubble test OSP<20cm H_2_O Audible sound	Take proper size PLMA Change the mask Deepen anaesthesia Ensure palatal pressure and proper fixation PPV Correction of malposition 1*
(6) Airway obstruction Inability to ventilate Bag slowto fill up	Severe epiglottic downfolding 4 Glottic/supraglottic compression 5 Cuff infolding 6 Reflexglottic closure 7	Increased PAP MMV Fiberoptic examination	Reinsertion with maintained laryngoscopy or jaw thrust 4* Air should be with drawn from the cuff 5*, 6* Take proper size PLMA, one size smaller may be tried for cuff infolding 5*, 6* Ensure correct cuff inflation pressures 5*, 6* Deepen anaesthesia or muscle relaxant^7^*
(7) • High PAP without apparent cause (Obesity, COPD)	Malposition^4^‐^6^ Light plane of anaesthesia	Fiberoptic examination Gagging Bronchospasm Laryngospasm	Correction of malposition 4*–6* Deepen anaesthesia
(8) • Singing patient	Inappropriate size of the mask Light anaesthesia Malposition 4‐6	Increased airway resistance Increased PAP Fiberoptic examination MMV test	Change mask Deepen anaesthesia Correction of malposition^4^*–6*
(9) • Abdominal distension	Gastric insufflation / gastric dilatation due to PPV with face mask prior to insertion of device Malposition^1^,^3^ Breach in the oropharyngeal seal	Visual assessment Fiberoptic examination	Gastric tube insertion and intermittent suction Correction of malposition^1^*,^3^* Change mask
(10) • Regurgitation through drain tube	Light plane of anaesthesia Head down position >30° Laparoscopic surgery Rule out aspiration	Fluid in the drain tube Increased PAP	Deepen anaesthesia Gastric tube insertion and intermittent suction
(11) • Laryngospasm	Rule out light plane of anaesthesia	Audible sound Excessive secretions	Deepen anaesthesia Suction
(12) • Bronchospasm	Rule out -Aspiration -Malposition 4‐6	Fiberoptic examination	Fiberoptic suction Correction of malposition^4^*–6* Bronchodilators

1 – 6 Malposition, Gum elastic bougie (GEB), Peak airway pressure (PAP)			
1* – 6* Specific Solutions, Positive pressure ventilation (PPV)			
COPD = Chronic obstructive pulmonary disease			

### II. Regurgitation and aspiration

Regurgitation of gastric contents may result in supracuff soiling of the mask and pulmonary aspiration with catastrophic results.^19^,^29^ This may be precipitated by activation of protective reflexes due to light plane of anaesthesia as greater depth of anaesthesia is required for insertion of PLMA as compared to cLMA.^30^

## Diagnosis

- Fluid seen in the airway /drain tube
- Intraoral examination
- Fiberoptic examination of the tracheo bronchial tree
- Sudden bronchospasm
- Haemodynamic instability
- Supracuff soiling of the mask on removal

### Management of Regurgitation^31^

- Leave the PLMA in situ
- Suction of the gastric tube and the drain tube
- Head down position and 100% oxygen should be administered
- Fiberoptic evaluation and suction
- Consider deepening level of anaesthesia and intubation of the patient fiberoptically via the PLMA

### III. Airway morbidity and Trauma

Airway morbidity and trauma may result from difficulty and multiple attempts at insertion, prolonged surgery without intracuff monitoring and improper size selection of the PLMA.^19^

## Diagnosis

- Coughing, gagging, retching stridor, hiccup, orbiting
- Audible noise, or as subtle increases in airway pressure orreductions in tidal volume
- Laryngospasm, bronchospasm, regurgitation, and aspiration

### Management 19,31

- Eliminating the source of stimulation
- Deepening anaesthesia
- Head down position and 100% oxygen should be administered
- Bronchodilators or high concentration of volatileagent

Many problems can be solved by proper patient and procedure selection, diagnosis and management of malpositions. Strategies to facilitate insertion by lateral/guided insertion techniques and maintaining adequate plane of anaesthesia may be helpful in improving first time and overall insertion success rates, correcting malpositions, overcoming difficult airway scenarios and prevention of regurgitation and pulmonary aspiration. Proper fixation of the device and monitoring cuff pressure in traoperatively especially during nitrous oxide based anaesthesia may bring down the incidence of airway morbidity.
